# Small RNA Sequencing of Sporadic Amyotrophic Lateral Sclerosis Cerebrospinal Fluid Reveals Differentially Expressed miRNAs Related to Neural and Glial Activity

**DOI:** 10.3389/fnins.2017.00731

**Published:** 2018-01-09

**Authors:** Rachel Waller, Matthew Wyles, Paul R. Heath, Mbombe Kazoka, Helen Wollff, Pamela J. Shaw, Janine Kirby

**Affiliations:** Department of Neuroscience, Sheffield Institute for Translational Neuroscience, University of Sheffield, Sheffield, United Kingdom

**Keywords:** amyotrophic lateral sclerosis, cerebrospinal fluid, small RNA sequencing, microRNAs, biomarker

## Abstract

Amyotrophic lateral sclerosis (ALS) is a clinical subtype of motor neurone disease (MND), a fatal neurodegenerative disease involving the loss of both the upper and lower motor neurones from the motor cortex, brainstem, and spinal cord. Identifying specific disease biomarkers would help to not only improve diagnostic delay but also to classify disease subtypes, monitor response to therapeutic drugs and track disease progression. miRNAs are small non-coding RNA responsible for regulating gene expression and ultimately protein expression and have been used as biomarkers for many cancers and neurodegenerative disorders. Investigating the detection of miRNAs in cerebrospinal fluid (CSF), the fluid that bathes the central nervous system (CNS) is a prime target for identifying potential biomarkers for ALS. This is the first study to investigate the expression of miRNAs in the CSF of ALS patients using small RNA sequencing. We detected 11 differentially expressed miRNAs in the CSF of sporadic ALS (sALS) patients related to neural and glial activity. Additionally, miRNAs involved in glucose metabolism and the regulation of oxidative stress were also identified. Detecting the presence of potential CSF derived miRNA biomarkers in sALS could open up a whole new area of knowledge to help gain a better understanding of disease pathophysiology. Additionally, with further investigation, the tracking of CSF miRNA over the disease course could be used to follow the disease progression and monitor the effect of novel therapeutics that could be personalized to an individual disease phenotype.

## Introduction

Amyotrophic lateral sclerosis (ALS) is the most common clinical subtype of motor neurone disease (MND), a fatal neurodegenerative disease involving the loss of both the upper and lower motor neurones from the motor cortex, brainstem, and spinal cord. Motor neurone loss in ALS leads to progressive muscle wasting and weakness, with eventual death, in the majority of cases due to respiratory failure, within 2–3 years from symptom onset.

Diagnosing ALS can be difficult, with the disease showing substantial clinical and prognostic heterogeneity (Benatar and Wuu, 2012). Many ALS symptoms are typical of other similarly presented diseases such as myopathy, neuropathy, structural spinal disorders and myasthenia gravis (MG) (Ghasemi, 2016). Therefore, identifying specific disease biomarkers would help to not only diagnose ALS but also help to classify disease subtypes, monitor a patient's response to therapeutic drugs and track disease progression.

Our recently published paper shows the potential use of serum-based microRNAs (miRNAs) as biomarkers of ALS disease progression (Waller et al., 2017). miRNAs are small non-coding RNAs responsible for regulating gene expression and ultimately protein expression. Around 70% of miRNAs are expressed in the brain, with many being brain-specific or brain-enriched (Muller et al., 2014). Investigating miRNAs in cerebrospinal fluid (CSF), the fluid that bathes the central nervous system (CNS) is a prime target biosample for identifying potential biomarkers for ALS. miRNAs present in CSF are increasingly being used as robust prognostic and diagnostic biomarkers in cancer (Drusco et al., 2015; Akers et al., 2017). In addition miRNA dysregulation in the CSF has frequently been reported in a range of neurological disorders including multiple sclerosis (MS) (Bergman et al., 2016; Quintana et al., 2017), Alzheimer's Disease (AD) (Denk et al., 2015; Sorensen et al., 2016), Parkinson's Disease (PD) (Gui et al., 2015; Marques et al., 2016) and ALS (Freischmidt et al., 2013; Benigni et al., 2016). Plasma, urine and saliva have been investigated as potential sources of disease biomarkers, however, miRNA biomarkers identified in the CSF may reflect changes in the degenerating cells, or could be markers reflecting pathophysiological changes in the brain, representing a more sensitive indicator of brain pathology than those present in blood or other biofluids.

Only three previous publications have investigated miRNA expression in CSF from patients with AD and PD using small RNA sequencing (Burgos et al., 2013, 2014; Yagi et al., 2017). This is the first study to investigate miRNA expression in CSF derived from ALS patients using small RNA sequencing. We identify several differentially expressed miRNAs in sporadic ALS (sALS) patient CSF samples compared to control subjects. Following qPCR validation, we discuss the challenges of these experiments in CSF studies. Overall, identifying the presence of potential miRNA biomarkers in sALS CSF samples is a promising tool that, with further investigation, could open up a whole new area of knowledge to help gain a better understanding of the disease.

## Materials and methods

**Figure 1:**
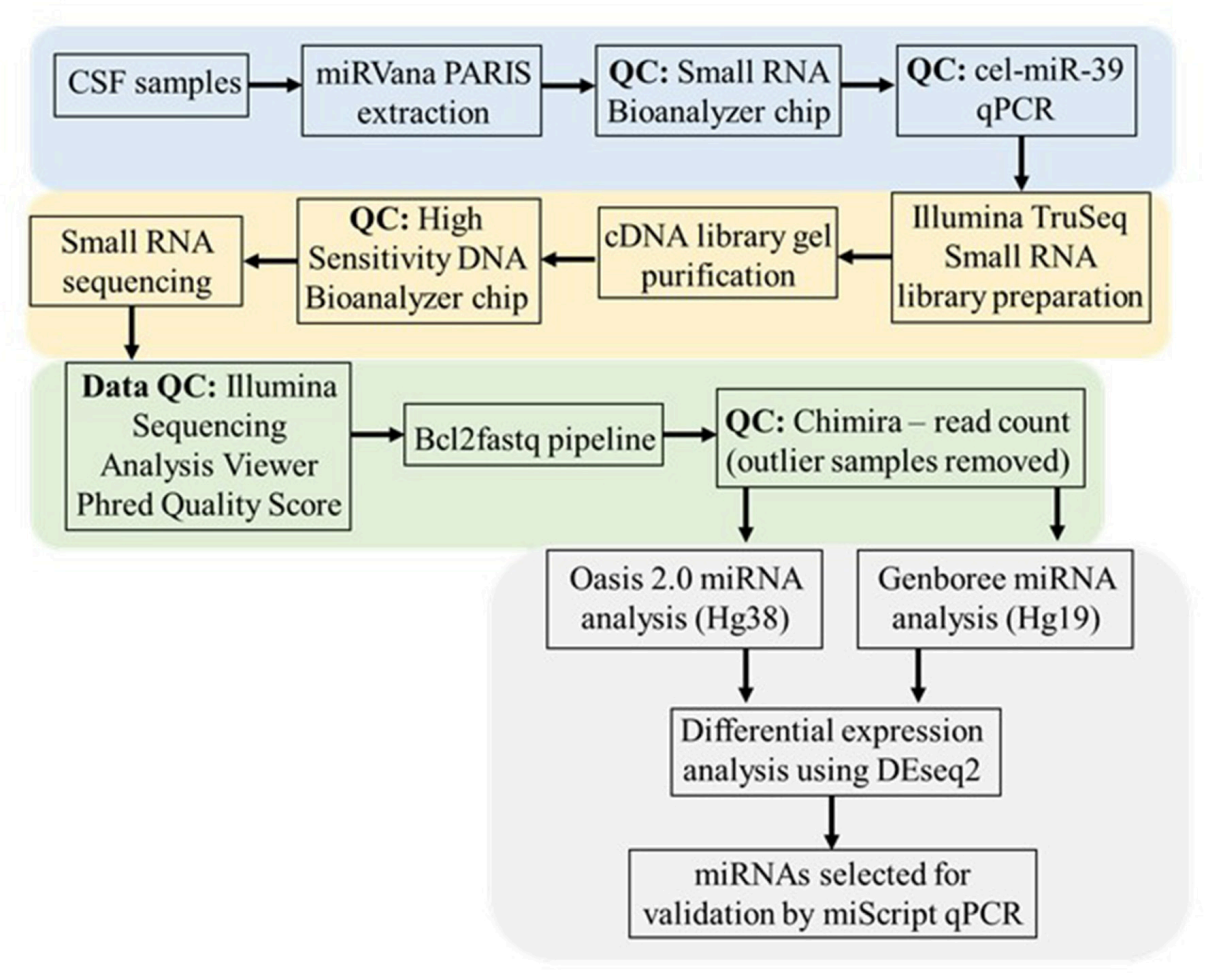
CSF small RNA sequencing methodology and data analysis workflow. This workflow begins with RNA extraction from CSF samples using the miRVana PARIS RNA extraction kit. The proceeding quality control (QC) steps performed prior to small RNA sequencing include the running of a small RNA Bioanalyzer chip and qPCR for the cel-miR-39 spike-in. The extracted RNA was subjected to Illumina TruSeq library preparation and gel purification and subsequent QC steps prior to small RNA sequencing. Initial sequencing data was put through the Illumina Sequencing Analysis Viewer to establish the Phred Quality score (Q > 30). Following this the raw .bcl generated image files were de-multiplexed and converted to .fastq text files where initial QC testing was carried out in Chimira to establish the distribution of read counts amongst each sequenced sample and identify any outlier samples. Both Oasis 2.0 and Genboree platforms were used to identify the specific miRNA content in each sample prior to using DEseq2 in both platforms to determine differential expressed miRNAs in sALS patients compared to control subjects (*p* ≤ 0.05). Based on comparing the commonly expressed and uniquely significant miRNA between each analysis platform individual miRNAs were chosen for validation via miScript qPCR.

The pipeline methods are depicted as a flowchart in Figure 1.

### Sample collection and patient data

CSF was collected at the Royal Hallamshire Hospital Sheffield, and all laboratory work was conducted at the Sheffield Institute for Translational Neuroscience (SITraN). This study (12/YH/0330) was carried out in accordance with the recommendations of NRES Committee Yorkshire & The Humber - Sheffield with written informed consent from all subjects. All subjects gave written informed consent in accordance with the Declaration of Helsinki. The protocol was approved by the NRES Committee Yorkshire & The Humber - Sheffield. CSF samples were obtained by lumbar puncture, held on ice and processed within 1 h by centrifugation at 800 g for 5 min to pellet cellular debris. The supernatant was removed, snap frozen and stored in liquid nitrogen prior to RNA extraction. We obtained CSF samples from the following three groups: sALS (*n* = 32), healthy controls (*n* = 10), neurological disease controls; multiple sclerosis (MS) patients (*n* = 6). Due to the limited availability of CSF samples obtained from healthy control subjects these were combined with CSF samples obtained from a group of neurological disease control subjects (MS Patients) for all downstream analyses forming a “combined control” cohort (*n* = 16), see results section for justification. An overview of the sample cohort is included in Table 1.

**Table 1:** Patient cohort.

	**sALS (*n* = 32)**	**Headache/migraine controls (*n* = 10)**	**MS (*n* = 6)**	**Combined Controls (*n* = 16)**
Mean age (range)	61.3 (40–84)	53.9 (37–81)	58.0 (54–69)	55.3 (37–81)
Gender (male:female)	21:11	3:7	3:3	6:10

*sALS, sporadic amyotrophic lateral sclerosis*.

### RNA extraction and quality control qPCR

Total RNA was extracted from 1 ml of CSF from each subject using the miRVana PARIS kit (Thermo Fisher Scientific) according to the manufacturer's instructions following the Total RNA Isolation Procedure with minor modifications; (1) As the yield of miRNAs in CSF was expected to be lower than other biofluid samples, 2 μl of glycogen (20 μg/μL) was added to each sample prior to total RNA extraction to enhance the binding of RNA to the column (McAlexander et al., 2013). (2) To control for variations of RNA extraction, each sample was spiked with 5 μl of 5 nM synthetic Caenorhabditis elegans microRNA 39 (Syn-cel-miR-39) (Qiagen).

Following extraction, it was not possible to quantify the miRNA concentration of CSF samples using the Nanodrop therefore, equivalent CSF volumes were used as input for the RNA extraction (Muller et al., 2014). Small RNA Bioanalyzer (Agilent) chips were used to identify the presence of miRNAs in the extracted samples (Supplementary Figure 1A). The Qiagen miScript PCR System was used to measure the recovery of small RNAs in each of the samples according to the cel-miR-39 spike-in concentration. Briefly, 5 μl of extracted RNA was reverse transcribed using the miScript II Reverse Transcription (RT) kit according to manufacturer's instructions (37°C for 60 min, 95°C for 5 min, followed by a 5 min incubation on ice). cDNA was diluted 5-fold in RNase-free water and added to the miScript qPCR reaction mix containing 2x QuantiTect SYBR Green PCR master mix (MM), universal primer and a specific cel-miR-39 miRNA primer assay. Amplification was performed using a CFX384 BioRad Real-Time PCR System with a denaturation step at 95°C for 15 mins, followed by 40 cycles at 94°C for 15 s, 55°C for 30 s and 70°C for 30 s.

### Small RNA-sequencing

RNA was prepared for small RNA-sequencing using the Illumina TruSeq Small RNA library preparation kit following a modified protocol as described in Burgos et al. (2013). Briefly, T4 RNA ligase was used to ligate RA5 and RA3 RNA oligonucleotides to the 5′ and 3′ ends of miRNA within 5 μl of extracted total RNA respectively. Adapter-ligated RNA was reverse transcribed to generate a cDNA library and amplified following 15 cycles of PCR (denaturation at 98°C for 30 s the products were amplified following 15 cycles at 98°C for 10 s, 60°C for 30 s, 72°C for 15 s and then 72°C for 10 min) incorporating individual barcodes to each sample to enable the pooling and loading of multiple samples onto the Illumina HiScanSQ. Individual cDNA library products of 140–160 bp were isolated on a 6% TBE PAGE gel (Supplementary Figure 1B). The quality of the each generated small RNA-sequencing cDNA library was assessed using High Sensitivity DNA chips on the Agilent 2100 Bioanalyzer (Supplementary Figure 1C). For cluster generation 1000 pM of each cDNA library from 12 samples (8 sALS and 4 controls) were pooled together and loaded onto two lanes of the flow cell at 20 pM concentration per lane. Each sample pool was loaded across two lanes of the flow cell to maximize the number of mapped reads (50 bp single read). 1% PhiX was spiked into each lane of the flow cell as a sequencing control. We processed our samples using the Illumina TruSeq SBS Kit (v3) for 51 cycles of sequencing and for 7 cycles of the indexing read.

### Sequencing data analysis

Initial quality control (QC) testing of post-sequencing data was carried out using the Illumina Sequencing Analysis Viewer to identify the Illumina Phred quality score (>Q30) and pass filter (PF) reads. Illumina sequencers perform an internal quality filtering procedure called chastity filter, and reads that pass this filter are called PF removing the least reliable clusters from the image analysis results. Sequence data were converted from the generated image.bcl files to raw, de-multiplexed sequencing data in the.fastq text file format using the Illumina developed bcl2fastq pipeline. Raw sequencing data was deposited at NCBI's Gene Expression Omnibus (accession number GSE105811). Further QC testing of the raw data was completed using Chimira, a web-based system used for miRNA analysis from small RNA-sequencing data (Vitsios and Enright, 2015). Briefly, the 3′ adapter sequences were trimmed and the overall miRNA reads counted. Following QC, the individual sample data were uploaded into two independent online software packages designed for analyzing in detail small RNA-sequencing data; Oasis 2.0 (Love et al., 2014; Capece et al., 2015) and Genboree (Riehle et al., 2012; Coarfa et al., 2014; Subramanian et al., 2015). Specific trimming tools in each of these packages enabled the removal of the Illumina TruSeq 3′ adapter (TGGAATTCTCGGGTGCCAAGG) sequence from each sample's.fastq file and the read size filtered (15–32 nt) with low abundance reads (< 5 reads) discarded. All remaining reads were mapped to the Human genome (Oasis 2.0: hg38, Genboree: Hg19) and miRBase_v21. Both packages were then used to calculate the differential miRNA expression using DESeq2 between control and sALS samples. The DESeq2 method is used for differential analysis of count data, using shrinkage estimation for dispersions and fold changes to improve stability and data interpretation (Love et al., 2014). The method is based on negative binomial distribution, with custom fit for variance-mean dependence (Anders and Huber, 2010). The DESeq2 statistical analysis accounts for small replicate numbers, discreteness, large dynamic range of data and the presence of outliers (Love et al., 2014). Spearman's rank correlation coefficient was used to assess the relationship between age and miRNA read count on both Genboree and Oasis generated data, across the 15 candidate miRNAs. Additional non-parametric Mann-Whitney tests were used to assess the differences between miRNA read counts and gender.

### qPCR validation

For validation, individual cDNA samples from the initial QC testing of extracted CSF samples were directly subjected to a pre-amplification step using the miScript PreAMP PCR kit and specific validation primer mixes. The procedure was carried out according to the manufacturer's protocol with a denaturation step at 95°C for 15 min, followed by 12 cycles at 94°C for 30 s and 60°C for 3 min. 5 μl of diluted pre-amplified product was used according to the manufacturer's protocol, with miScript SYBR Green PCR Kit and individual miScript Primer Assay for qPCR on the CFX384 BioRad Real-Time PCR System following the same amplification steps as carried out for the QC cel-miR-39 qPCR. Samples were measured in duplicate. Relative expression levels of miRNAs were calculated using the comparative CT (2-ΔΔCT) method for specific miScript validation experiments, where CT is cycle threshold, and ΔCt = CT (miRNA) − CT (reference miRNA). Reference/normalization miRNAs were chosen based on their stable expression across sample groups demonstrated in the small RNA sequencing data analysis and through previously published literature. These 7 reference/normalization candidates (cel-miR-39, RNU6, miR-204-5p, miR-10a-5p, miR-10b-5p, miR-30a-5p, SNORD95) were first investigated by qPCR to ascertain stability across the investigated samples prior to the subsequent candidate miRNA qPCR validation. All statistical analysis between two comparison groups was carried out using the unpaired two-tailed *t*-tests in GraphPad Prism version 7.

## Results

### Individual sample quality control testing–pre-sequencing

**Figure 2:**
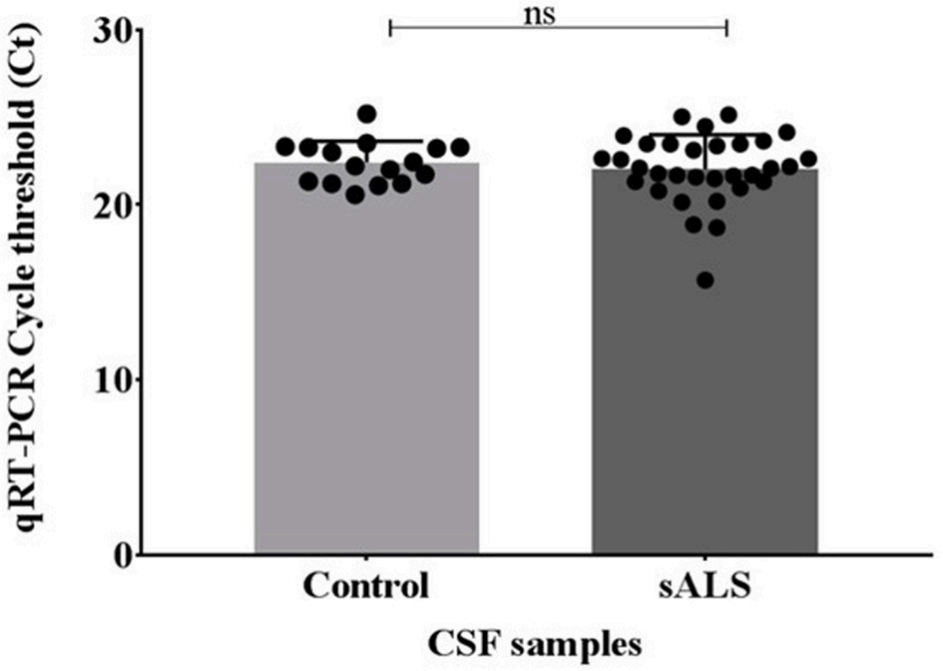
miRNA recovery of cel-miR-39 after miRVana PARIS extraction using Qiagen miScript qPCR. Ct values were compared across the control and sALS samples where the lowest Ct value indicates the highest amount of miRNA recovery. No significant difference in cel-miR-39 levels was seen between control and sALS samples. Ct, cycle threshold; CSF, cerebrospinal fluid; sALS, sporadic amyotrophic lateral sclerosis; ns, non-significant.

Pre-sequencing extracted samples were analyzed via qPCR for the cel-miR-39 spike-in. No significant difference was identified in the levels of cel-miR-39 between the control and sALS extracted CSF samples and therefore all samples were viable for small RNA sequencing (Figure 2).

### Individual sample quality control testing–post-sequencing

**Figure 3:**
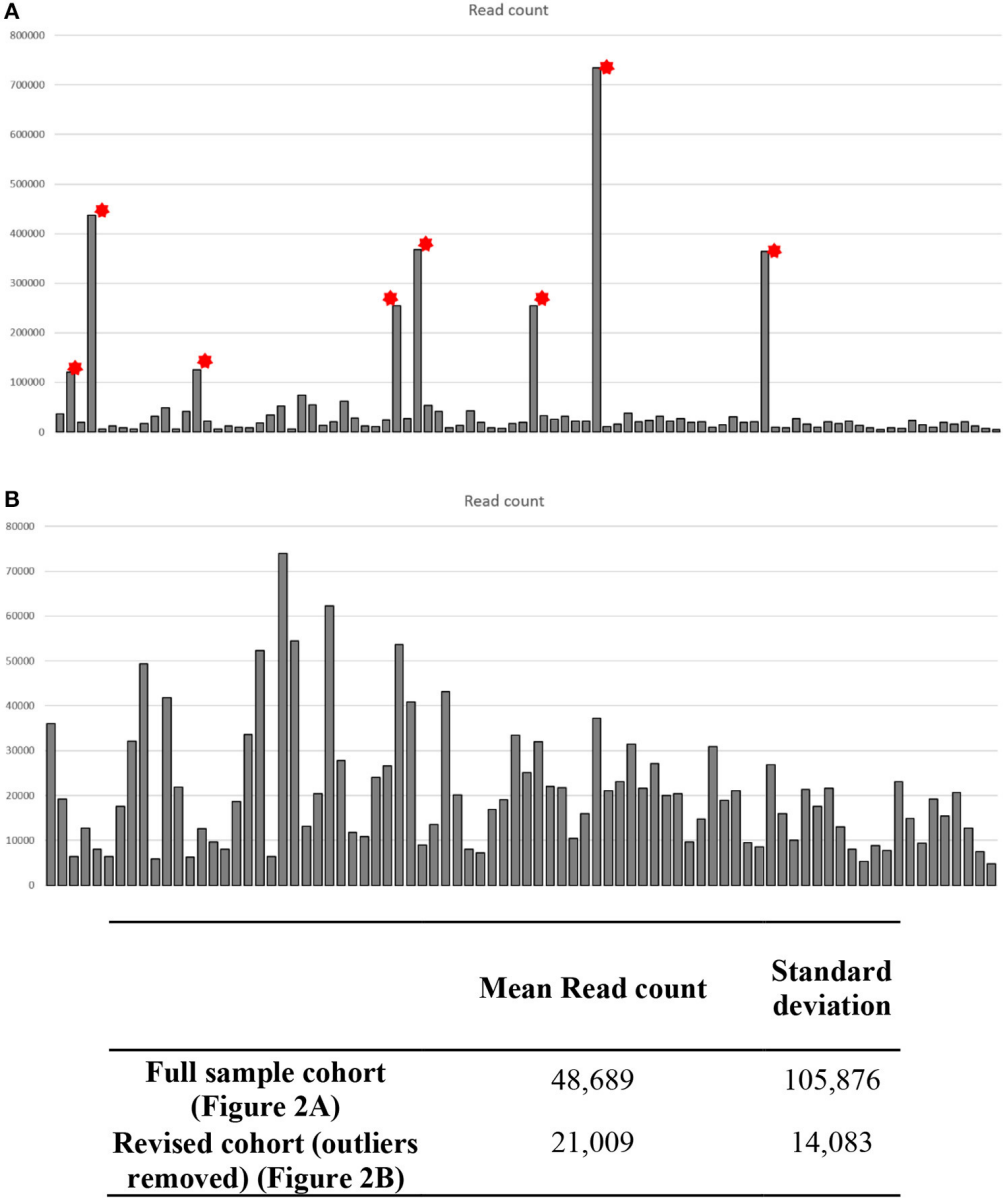
Quality control data analysis across CSF small RNA sequenced samples. Each sample was ran in duplicate across two lanes of a sequencing flow cell. Chimira was used to provide an overall assessment of the miRNA expression content in each sequenced sample across the two lanes. **(A)** Eight samples showed read counts that were considerably higher than the remaining 82 samples (^*^ outlier samples), these outlier samples would skew any differential expression analysis. **(B)** Upon removal of the 8 outlier samples each sample read count is now comparable for differential expression analysis.

Initial post-sequencing QC analysis was carried out using the Illumina Sequencing Analysis Viewer and Chimira. Each sample was run in duplicate across two lanes of the flow cell to maximize the read coverage. Using the methodology parameters, we got an average of 132–208 clusters per mm^2^ per flow cell lane. The Q30 scores remained above 97.5% throughout the sequencing run with an average of 3,202,323 (658,761–10,348,286) combined PF reads per sample identified. Chimira uses inputted sequences and maps these against miRBase_v21 in order to decipher the miRNA expression content in the input samples. Chimira identified a spread of miRNA mapped read counts ranging from 4,809 to 734,672 across the samples. In doing this 8 outlier samples (Figure 3A red asterisk) were easily identified, this accounted for 3 sALS cases and 3 control subjects. These particular samples were removed to reveal more comparable samples ranging from 4,809 to 74,004 read counts (Figure 3B). These samples were taken forward for further interrogation in both Oasis 2.0 and Genboree analysis platforms.

### Oasis 2.0 and genboree miRNA expression profiling

We carried out downstream analysis using two bioinformatics platforms Oasis 2.0 and Genboree. When analyzing the data from all the sequenced CSF samples simultaneously we detected 179 different miRNAs expressed at least once via Oasis 2.0 and 274 different miRNAs via Genboree. The top 30 most abundant miRNAs as identified by Oasis 2.0 and Genboree from all the CSF samples are presented in Table 2. The complete list of known miRNAs identified by both platforms for all analyzed samples is provided in Supplementary Tables 1 and 2.

**Table 2:** Top 30 most abundant miRNAs identified in human CSF samples analyzed by Oasis 2.0 and Genboree.

**Rank**	**Oasis 2.0**	**Base Mean read count**	**Rank**	**Genboree**	**Base Mean read count**
1	cel-miR-39-3p	5,5561	1	hsa-miR-204-5p	5,448
2	hsa-miR-204-5p	2,951	2	hsa-miR-143-3p	3,882
3	hsa-miR-143-3p	2,111	3	hsa-miR-10b-5p	2,327
4	hsa-miR-125b-2-3p	1,455	4	hsa-miR-486-5p	2,139
5	hsa-miR-10b-5p	1,202	5	hsa-miR-181a-5p	1,702
6	hsa-miR-181a-5p	906	6	hsa-miR-125b-2-3p	1,424
7	hsa-miR-92a-3p	605	7	hsa-miR-92a-3p	1,190
8	hsa-miR-27b-3p	604	8	hsa-miR-10a-5p	1,091
9	hsa-miR-10a-5p	561	9	hsa-miR-27b-3p	1,047
10	hsa-miR-30a-5p	514	10	hsa-miR-30a-5p	993
11	hsa-miR-125b-5p	464	11	hsa-miR-22-3p	882
12	hsa-miR-22-3p	460	12	hsa-let-7f-5p	704
13	hsa-miR-486-5p	377	13	hsa-miR-26a-5p	589
14	hsa-let-7f-5p	369	14	hsa-miR-21-5p	566
15	hsa-miR-26a-5p	361	15	hsa-let-7a-5p	487
16	hsa-miR-21-5p	304	16	hsa-miR-92b-3p	477
17	hsa-let-7a-5p	245	17	hsa-miR-423-5p	460
18	hsa-miR-92b-3p	242	18	hsa-miR-30d-5p	241
19	hsa-miR-30d-5p	127	19	hsa-miR-101-3p	222
20	hsa-miR-101-3p	123	20	hsa-miR-155-5p	205
21	hsa-miR-155-5p	117	21	hsa-let-7i-5p	203
22	hsa-let-7i-5p	105	22	hsa-miR-148a-3p	189
23	hsa-miR-148a-3p	103	23	hsa-miR-151a-3p	169
24	hsa-miR-9-5p	93	24	hsa-miR-9-5p	164
25	hsa-miR-151a-3p	89	25	hsa-miR-191-5p	155
26	hsa-miR-30e-5p	80	26	hsa-miR-30e-5p	154
27	hsa-miR-191-5p	80	27	hsa-miR-16-5p	152
28	hsa-miR-16-5p	78	28	hsa-miR-146a-5p	148
29	hsa-miR-28-3p	77	29	hsa-let-7c-5p	143
30	hsa-miR-146a-5p	74	30	hsa-miR-28-3p	142

### miRNAs are differentially expressed in sALS patients

Initial data analysis was carried out to investigate whether any statistical differences in miRNA expression existed between the two control groups. There were only 4 miRNAs identified as significantly different between the neurological disease controls (MS cases) and healthy control cases (headache/migraine cases); miR-142-5p, let-7g-5p, miR-150-5p, and miR-4486 (Supplementary Tables 1 and 2). However, neither let-7g-5p nor miR-4486 were identified as significantly different between either control group comparisons with sALS patients. miR-142-5p and miR-150-5p were identified as significantly downregulated in the sALS cases compared to the healthy control group and a non-significant down regulation in these two miRNAs was also identified in sALS cases when compared to the neurological disease controls (MS group). Consequently, this suggests that analyzing the data by including the neurological disease control cases (MS cases) with the healthy control subjects maintained the differences in miRNA expression and helped to increase control cohort size and significance and therefore all further analysis was carried out combining the two control groups, known as the “combined control” cohort.

**Figure 4:**
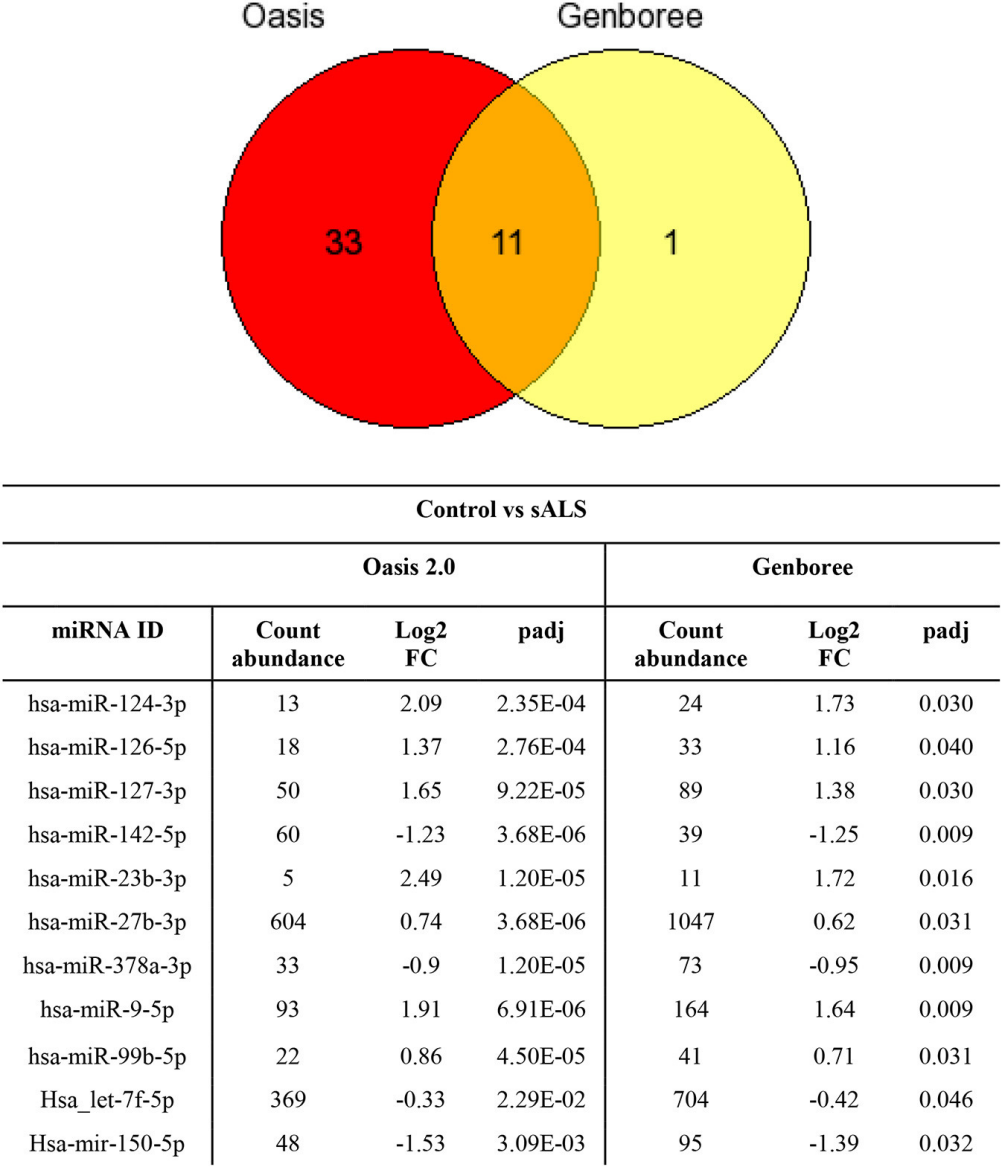
Comparing the significantly expressed miRNAs identified between the two platforms, 11 miRNAs are common between the two as listed. There are more significantly expressed miRNAs identified by Oasis 2.0 platform. Only those miRNAs that have read counts above 50 were considered for validation.

The sample data from the combined control group and sALS patient group were subjected to miRNA differential expression analysis via both Genboree and Oasis 2.0 platforms using DeSeq2. Significant differential expression was determined in sALS patients compared to control subjects. Results were filtered at corrected *p* < 0.05 and normalized mean >5 mapped reads. Eleven significant miRNAs were determined in both analysis platforms (Figure 4) with an additional 33 significant miRNA changes determined specifically by Oasis 2.0 and one additional significant miRNA change determined specifically by Genboree. Only those significant miRNA changes with read abundance calls >50 were considered for validation (7/11 common miRNAs) with miR-124 also added to the candidate list due to its previously established role in the brain (Cheng et al., 2009; Maiorano and Mallamaci, 2010). An additional 7 significantly expressed miRNAs as determined by Oasis 2.0 analysis were also considered for validation based on their mean read abundance being >50 and the Genboree analysis for those particular miRNA being just out of significance, or due to the miRNAs having an already established role in the brain/neurological disease. In total 15 miRNAs were chosen for validation as outlined in Table 3 and their function summarized in Table 4. Analysis of miRNA read count data showed no significant correlation of any of the 15 candidate miRNAs with increasing age in both Genboree and Oasis (data not shown). Additionally no difference in miRNA read count was identified between male and female subjects (data not shown).

**Table 3:** Validation miRNAs.

**miRNA**	**Count abundance**		**log2FoldChange (control vs. sALS)**		**Increase/Decrease**
	**Oasis 2.0**	**Genboree**	**Oasis 2.0**	**Genboree**	**Control vs. sALS**
miR-124-3p	13	24	2.09	3.17	Increased
miR-127-3p	50	89	1.65	1.38	Increased
miR-143-3p	2111	3882	0.58	0.48	Increased
miR-125b-2-3p	1455	1424	0.58	0.62	Increased
miR-9-5p	93	164	1.91	1.64	Increased
miR-27b-3p	604	1047	0.74	0.62	Increased
miR-486-5p	377	2139	−1.13	−0.85	Decreased
Let-7f-5p	369	704	−0.33	−0.42	Decreased
miR-16-5p	78	152	−0.76	−0.77	Decreased
miR-28-3p	77	142	−0.58	−0.59	Decreased
miR-146a-5p	74	148	−0.85	−0.82	Decreased
miR-150-5p	48	95	−1.53	−1.39	Decreased
miR-378a-3p	33	73	−0.9	−0.95	Decreased
miR-142-5p	30	39	−1.23	−1.25	Decreased
miR-92a-5p	605	1190	−0.52	−0.54	Decreased

*Values in blue indicate significantly differentially expressed miRNAs that were identified by Oasis 2.0 only. Values in yellow indicate significantly differentially expressed miRNAs that were identified by both Oasis 2.0 and Genboree. Results were filtered at corrected p-value < 0.05. sALS, sporadic amyotrophic lateral sclerosis*.

**Table 4:** A summary of the candidate validation miRNAs and their known function.

**miRNA**	**Description**	**References**
miR-124-3p	A highly expressed miRNA in the CNS	Cheng et al., 2009; Meza-Sosa et al., 2014
	Involved in maintaining neuronal cell identity	Maiorano and Mallamaci, 2010
	Involved in maintaining synaptic plasticity	Rajasethupathy et al., 2009
	Found in the brain of ALS mice at late stage disease, with miR-9 and miR-125 linked to neural activity	Marcuzzo et al., 2015
	A suggestive marker of brain injury in a rat stroke model	Laterza et al., 2009
miR-127-3p	Linked to neuronal loss, neurodegeneration and apoptosis in primary cultured spinal neurones	He et al., 2016a
miR-143-3p	Found to have a negative role on myoblast differentiation with increasing levels associated with muscle denervation	Du et al., 2016
	Increased levels found in serum of sALS patients	Waller et al., 2017
	Increased levels linked to an anti-proliferative and pro-apoptotic role	Borralho et al., 2009; He et al., 2016b; Li et al., 2017
	Found to be induced in rat neurones, associated in the regulation of glucose metabolism in experimental ischemic injury	Zeng et al., 2017
miR-125b-2-3p	A known regulator of glia, associated with astrogliosis	Smirnova et al., 2005; Pogue et al., 2010
	Associated with microgliosis and linked to neuroinflammation in ALS	Parisi et al., 2013
	Found in the brain of ALS mice at late stage disease along with miR-124 and miR-9 linked to corticospinal tract degeneration in ALS	Marcuzzo et al., 2015
	Circulating miR-125b and miR-9 was downregulated with miR-9 in an APP/PS1 transgenic mouse model of AD. A potential biomarker	Hong et al., 2017
miR-9-5p	A brain enriched miRNA	Meza-Sosa et al., 2014
	Found in the brain of ALS mice at late stage disease with miR-124 and miR-125 linked to neural activity	Marcuzzo et al., 2015
	Circulating miR-9 and miR-125b was downregulated in an APP/PS1 transgenic mouse model of AD. A potential biomarker	Hong et al., 2017
miR-27b-3p	A highly expressed miRNA in the brain linked to bipolar disorder and schizophrenia	Moreau et al., 2011
miR-486-5p	Dysregulated in many human cancers, acting as a tumor suppressor	Oh et al., 2011; Peng et al., 2013
miR-16-5p	Reduced levels identified in a murine model of early-onset AD, linking reduced levels with increased levels of amyloid precursor protein (APP)	Liu et al., 2012
	A neuroprotective role in prion infected mice	Majer et al., 2012
miR-28-3p	Circulating miR-28-3p was upregulated in an APP/PS1 transgenic mouse model of AD. A potential biomarker	Hong et al., 2017
miR-146a	An immune-mediated miRNA, a key regulator of the innate immune response increased in active MS lesions in humans	Junker et al., 2009
miR-150-5p	An immune-mediated miRNA, circulating miR-150-5p increased in CSF of MS patients and serum of MG patients	Punga et al., 2015; Bergman et al., 2016
miR-378a-3p	Involved in myogenesis and regulation of skeletal muscle growth, promoting myoblast differentiation and inhibiting proliferation	Wei et al., 2016
miR-142-5p	An important regulator of cell survival. In a neuronal ischemic injury cell model miR-142-5p was induced by neurones. Inhibition of miR-142-5p reduced cell injury and oxidative stress in the model by upregulating the Nrf2/ARE signaling pathway	Wang et al., 2017
miR-92a-3p	miR-92a-3p linked to white matter impairment and post-stroke depression	He et al., 2017

*CNS, central nervous system; AD, Alzheimer's disease; sALS, sporadic amyotrophic lateral sclerosis; MS, multiple sclerosis; MG, myasthenia gravis*.

### Small RNA sequencing validation

Using the miScript qPCR system attempts were made to validate the small RNA sequencing findings. However, there are many challenges of using qPCR for this application as highlighted by other studies (Metpally et al., 2013). Despite statistically significant differences in miRNA expression identified by small RNA sequencing, qPCR validation failed to replicate this. However, 7/15 candidates show the same directional change in the samples as identified in the initial sequencing study (Supplementary Figure 2).

## Discussion

We began our studies with the focus to identify specific miRNA biomarkers present in the CSF of sALS patients. Our aim was to use small RNA sequencing to investigate differentially expressed miRNAs in the CSF of sALS patients compared with control subjects. Overall, this novel study involved the optimization of methods involved in RNA extraction, small RNA sequencing library preparation and relevant quality control testing of samples. Subsequently we assessed the use of various different data analysis software packages in order to determine a pipeline that allowed us to carry out miRNA differential expression analysis between control subjects and sALS patients. We tested the robustness of the sequencing data by attempting to validate the miRNA expression with a second type of detection, using Qiagen miScript qPCR probes. Candidate miRNAs for validation were chosen from the significantly expressed miRNAs showing an average abundance read count of more than 50 across the patient/control groups or based on previous literature identifying their presence in the nervous system. Around half of the candidate miRNAs showed the same directional change as identified in the sequencing study, which however failed to reach statistical significance. Whilst the candidate miRNAs were not statistically validated due to technical issues, these miRNAs have been implicated in the literature.

Our analysis revealed the upregulation of a number of miRNA, specifically associated with a known CNS presence; miR-124-3p, miR-9-5p, miR-125b-2-3p, and miR-27b-3p. miR-124 is one of the most highly expressed miRNAs in the CNS (Cheng et al., 2009; Meza-Sosa et al., 2014) and has been associated with maintaining neuronal cell identity (Maiorano and Mallamaci, 2010) and synaptic plasticity (Rajasethupathy et al., 2009). miR-9 is an evolutionary-conserved brain enriched miRNA (Meza-Sosa et al., 2014), while miR-125b is a known regulator of glia (Smirnova et al., 2005) and has been associated with astrogliosis (Pogue et al., 2010) and microgliosis linked to neuroinflammation in ALS (Parisi et al., 2013). miR-27b-3p is highly expressed in the brain and has been linked to bipolar disorder and schizophrenia (Moreau et al., 2011).

The upregulation of miR-9, miR-124 and miR-125b have been found in the brain of ALS mice at late disease stage (Marcuzzo et al., 2015), with miR-124 and miR-9 linked to neural activity and miR-125b linked to the corticospinal tract degeneration in ALS (Marcuzzo et al., 2015). Previous studies have suggested that a failure of cell cycle regulation could lead to the post-mitotic motor neurones attempting to re-enter the cell cycle, leading to cell death associated with the disease. Therefore, increased expression of these three particular miRNAs has been suggested to result from brain injury due to neurological disease. Consequently, the increased expression of miR-124-3p and miR-9-5p in sALS patient CSF could reflect neuronal injury and cell death, while an increase in miR-125b may represent a by-product of neuroinflammation and gliosis occurring in the brain during disease (Parisi et al., 2013).

An increase in the plasma concentration of miR-124 was seen 8 h after surgery to produce brain injury in a rat stroke model and peaked at 24 h, suggesting that circulating miR-124-3p is a marker of brain injury (Laterza et al., 2009). In stroke, a series of pathological changes results in damage to brain cells and it is suggested these products of cellular damage are released into circulation. Evidently, as ALS pathogenesis involves the death of motor neurones and increase in gliosis, the increased expression of both neural markers miR-124 and miR-9 and the glial marker miR-125b in the CSF could be used as a marker of brain injury, more specifically neuronal death and gliosis associated with the disease. Additionally, the highly expressed brain miRNA, miR-127-3p, was also increased in the CSF of our sALS patients. An increased expression of miR-127-3p has previously been reported in primary cultured spinal neurones leading to neuronal loss, neurodegeneration and neuronal apoptosis (He et al., 2016a).

Investigating whether the expression of these miRNAs continues to increase in the CSF over the disease course will be crucial as to the value of these CSF miRNAs as useful biomarkers of disease progression, and their potential as prognostic indicators.

Recently we reported the increased expression of miR-143-3p in sALS patient serum samples, suggesting its negative role on myoblast cell differentiation with increasing expression levels associated with muscle denervation (Waller et al., 2017). Likewise miR-143-3p was also increased in the CSF of sALS patients in the current study. An increased expression of miR-143-3p has been identified as playing a role in many different cancers and it's over expression linked to an anti-proliferative and pro-apoptotic role (Borralho et al., 2009; He et al., 2016b; Li et al., 2017). Therefore, increased expression of miR-143-3p in the CSF of sALS patients may be a by-product from dying motor neurones which could further contribute to motor neurone death associated with the disease. Additional functional studies would be required to investigate this further.

Another highlighted role of miR-143 is in the regulation of glucose metabolism. Following experimental ischemic injury, miR-143-3p is significantly induced in rat neurones, and this has a direct effect on glucose metabolism, suppressing glucose uptake and lactate production (Zeng et al., 2017). Equally, glycolysis enzymes hexokinase 2 (HK2), pyruvate kinase muscle isozyme M2 (PKM2) and lactate dehydrogenase A (LDHA) were also inhibited by experimental ischemic injury, with the over expression of miR-143-3p further inhibiting HK2, glucose uptake and lactate production (Zeng et al., 2017). HK2 is a direct target of miR-143-3p, hence restoring HK2 activity in miR-143-3p over expressing neurones rescued glucose uptake and lactate production (Zeng et al., 2017). Our current study also identified a down regulation of miR-142-5p, an important regulator of cell survival. In a cellular model of neuronal ischemia/reperfusion injury, miR-142-5p was induced in the hippocampal neurones. Inhibition of miR-142-5p reduced cell injury and oxidative stress in the model by upregulating the Nrf2/ARE signaling pathway (Wang et al., 2017). Evidently, the down regulation of miR-142-5p in our current study may work to oppose the upregulation of miR-143-3p in an attempt to regulate further neuronal loss and brain injury. Consequently, inhibiting miR-143-3p and increasing miR-142-5p expression during ischemic brain injury could help to protect neuronal cells. This represents a possible therapeutic measure in ALS where metabolic dysregulation and oxidative stress are considered to play a role in disease pathogenesis.

A number of miRNAs identified in the current study by small RNA sequencing were down- regulated in sALS patients compared to control subjects. However, any functional implication of this dysregulation in CSF circulating miRNAs in ALS remains to be investigated.

From the current literature, several possible roles/functions of these dysregulated miRNAs have been presented. For example miR-150-5p and miR-146a are immune-mediated miRNAs with circulating miR-150-5p increased in the CSF of MS patients (Bergman et al., 2016) and the serum of MG patients (Punga et al., 2015). miR-146a-5p, a key regulator of the innate immune response, was markedly increased in active MS lesions in humans (Junker et al., 2009). In contrast, the current study identified decreased expression of both miR-150-5p and miR-146a-5p in CSF samples of sALS patients. This may be a result of the samples being taken from patients at particular points in their disease where neuroinflammation was not at its highest, or perhaps these miRNAs may have distinct roles that are not yet known.

Additionally miR-378a-3p, which is known to be involved in myogenesis and regulation of skeletal muscle growth by promoting myoblast differentiation and inhibiting proliferation (Wei et al., 2016), is decreased in the CSF samples of sALS patients. Again, this may suggest an alternative currently unknown role of the miRNA, specifically within the brain/CSF environment, in comparison to the periphery.

Cancer related studies have identified the dysregulation of miR-486-5p in association with many human cancers (Chen et al., 2015), specifically demonstrating reduced expression linked to cancer development with studies identifying the role of miR-486-5p as a tumor suppressor (Oh et al., 2011; Peng et al., 2013). Therefore, the particular genes and molecular pathways targeted by miR-486-5p in its tumor suppressant role may play a part in cell death in neurodegenerative disease and further investigation is warranted.

A decrease in miR-16-5p was identified in a murine model of early-onset AD, which linked the reduced levels with increased levels of amyloid precursor protein (APP). Upon administrating miR-16-5p into the brains of these mice, APP levels reduced suggesting a neuroprotective role of miR-16-5p (Liu et al., 2012). Similarly, a neuroprotective role of miR-16-5p was identified in the brains from prion-infected mice. These mice presented an upregulation of miR-16-5p in early pre-clinical disease, with expression levels reducing with disease progression suggestive of a neuroprotective role of the miRNA (Majer et al., 2012). It would be interesting to determine whether in a similar way, miR-16-5p continues to decrease in sALS patients, corresponding to disease progression, and investigate the use of this miRNA as a potential prognostic biomarker.

With only three publications investigating miRNA expression using small RNA sequencing in CSF samples (Burgos et al., 2013, 2014; Yagi et al., 2017) there was limited information available to identify the best technical approach for this work. Consequently, many optimization studies preceded the final CSF small RNA sequencing presented in this report. Preliminary work identifying the most appropriate RNA extraction method to obtain an enriched level of miRNA had to be completed. In CSF the concentration of RNA is small and therefore the concentration of miRNA is even more limited and measuring miRNA concentration is problematic as previously reported (Buschmann et al., 2016). Prior investigations as to which library preparation kit was optimal for CSF samples were also completed. In addition, a number of further technical aspects to the methodology were assessed including: how to load each sample onto the sequencing flow cell (single or pooled), determining how many barcoded samples to load per lane of a flow cell, and the concentration of pooled samples to load to achieve optimal cluster density had to be established, along with the optimal method for data analysis.

Overall, considering all of the miRNAs that were most abundant in our CSF samples, our results were consistent with the other CSF small RNA sequencing publications where miR-204-5p was most highly abundant and most probably a CSF enriched miRNA (Burgos et al., 2013; Yagi et al., 2017). Also, our main findings were supported by the published literature describing detection of miRNAs including miR-486-5p, miR-143-3p miR-10a-5p, and miR-10b-5p represented within the top 10 most abundantly expressed miRNA in CSF (Burgos et al., 2013; Yagi et al., 2017). We therefore propose that the experimental strategy and analysis pipeline we followed is robust.

On the whole, there is little surprise that the differentially expressed miRNAs determined in this current study contrast with those we found in our previous work investigating serum based miRNAs as potential biomarkers of ALS (Waller et al., 2017). Not only do we use different technologies to assess miRNA expression, the implications of which are discussed in the current paper, but the two studies investigate very different biological fluids. As a miRNAs role is to rapidly and reversibly respond, their expression and ultimate function will be constantly changing and affected by many outside influences including a person's diet, lifestyle choice, etc. Clearly, since CSF is separated from blood circulation by the blood–brain barrier, the expression of CSF based miRNA will be influenced by different factors to those serum based miRNAs. For example, lower motor neuron axons reside outside of the CNS and so changes may be seen in the serum, which are not detected in the CSF. Undoubtedly, this will contribute to the contrast in miRNAs found in our studies and others (Burgos et al., 2013; Raoof et al., 2017).

Despite qPCR being used routinely as a methodology for validating gene expression microarray results, its ability to validate CSF based small RNA sequencing data is less reliable and an alternative method for validation is required. There are many reasons why qPCR may not be sufficient to validate small RNA sequencing data: (1) The sensitivity of qPCR is lower than in sequencing; (2) The miScript primer assays used in the validation qPCRs could lead to cross reactivity due to the small size of miRNAs. Hence any slight change in one or two nucleotides distinguishing between two separate miRNAs may not be picked up by qPCR; (3) The small size of miRNAs restricts the designing of optimized primers; (4) As identified in many miRNA expression studies, the normalization of data remains a controversial issue (Metpally et al., 2013; Muller et al., 2014; Benigni et al., 2016; Lusardi et al., 2017). In this study, we tried to approach the normalization issue in a methodical way by examining the sequencing read counts to identify the miRNAs that appeared constant across all samples analyzed. Prior to any downstream qPCR experiments the stability of 6 potential normalization miRNAs were investigated in our samples using the miScript qPCR system, and subsequently 2 normalization miRNAs were chosen for all the downstream qPCR investigations (cel-miR-39 and miR-30a-5p). Despite this, sequencing and qPCR are very distinct platforms with variability in sample preparation, normalization and reference gene selection contributing to these different results. Other factors that could influence study outcomes include insufficient sized cohorts, differences in sample reagents, differences in baseline miRNA levels due to age, disease progression or other epidemiological reasons (Lusardi et al., 2017). It is interesting to note that from all CSF based small RNA sequencing studies performed, none of the findings have been statistically validated using qPCR. Instead, the approach by many for profiling miRNA expression in CSF has been to use miRNA qPCR array plates, confirming findings using individual qPCR i.e., the same technology (Benigni et al., 2016; Akers et al., 2017; Lusardi et al., 2017; Quintana et al., 2017). Consequently, with future sequencing experiments, validation may be best suited to repeating the small RNA sequencing on an additional validation patient cohort to investigate whether the candidate miRNAs identified in the discovery cohort are replicated in the validation cohort. Inevitably, this approach would be limited by the availability of additional patient samples and the cost implications of running additional sequencing experiments.

Overall, the presence of differentially expressed miRNAs in the CSF of sALS patients compared to control subjects opens up a whole new field of understanding the pathophysiological basis of disease. Identifying the presence of a group of dysregulated miRNAs in the CSF of sALS patients could help in disease stratification and as prognostic indicators. In turn, looking at the expression of particular miRNAs during a patient's disease course could help to identify how well a particular therapy is working for that patient. In addition, this will help to identify sub groups of patients that may benefit/respond better to a specific therapy with the potential to open up the realm of personalized medicine for ALS.

## Author contributions

RW, MW, PH, PS, and JK: conceived and designed the experiments, and wrote the paper. MK, HW, and PS: consented and collected patient samples. RW and MW: performed the experiments. RW, MW, PH, and JK: analyzed the data.

### Disclosure statement

PS is supported as an NIHR Senior Investigator (NF-SI-0512-10082) and this work was supported by the Sheffield NIHR Biomedical Research Centre for Translational Neuroscience (IS-BRC-1215-20017).

### Conflict of interest statement

The authors declare that the research was conducted in the absence of any commercial or financial relationships that could be construed as a potential conflict of interest.

## Supplementary material

Supplementary Figure 1 Quality control testing of CSF small RNA sequencing library preparations. **(A)** A typical small RNA bioanalyzer trace of a miRVana PARIS extracted CSF sample (red, approximately 22nt) aligned with the small RNA ladder (blue). **(B)** A High sensitivity DNA bioanalyzer chip trace showing the CSF cDNA amplicon after 15 cycles of PCR amplification and the gel insert identifying the corresponding miRNA band in each sample (black box) during library preparation. **(C)** A High sensitivity DNA bioanalyzer chip trace showing the purified amplified CSF cDNA library construct, the 142 bp peak primarily containing mature microRNA generated from approximately 22 nt small RNA fragments. This sample is now ready for subsequent sample pooling and cluster generation.

Supplementary Figure 2 miRNA qPCR validation. From the candidate miRNAs identified as statistically significantly expressed in sALS patients using small RNA sequencing, 7/15 miRNAs showed a non-significant same directional change when investigated using qPCR **(A,E,G,I,L–N)**. The remaining 8/15 miRNAs showed very little change between sALS patients and control subjects using qPCR **(B–D,F,H,J,K,O)**.

Supplementary Table 1 Raw read counts generated by OASIS 2.0.

Supplementary Table 2 Raw read counts generated by Genboree.
