# Supplementary figures and images for: Small RNA Sequencing of Sporadic Amyotrophic Lateral Sclerosis Cerebrospinal Fluid Reveals Differentially Expressed miRNAs Related to Neural and Glial Activity
Source: Front Neurosci. 2018 Jan 9;11:731. doi: 10.3389/fnins.2017.00731 (PMC5767269; doi:10.3389/fnins.2017.00731)

Supplementary Figure 1

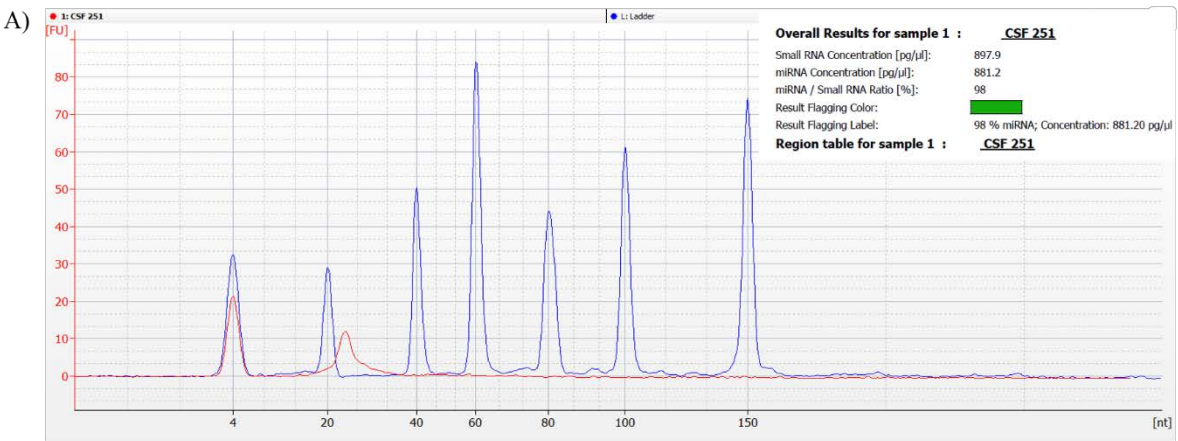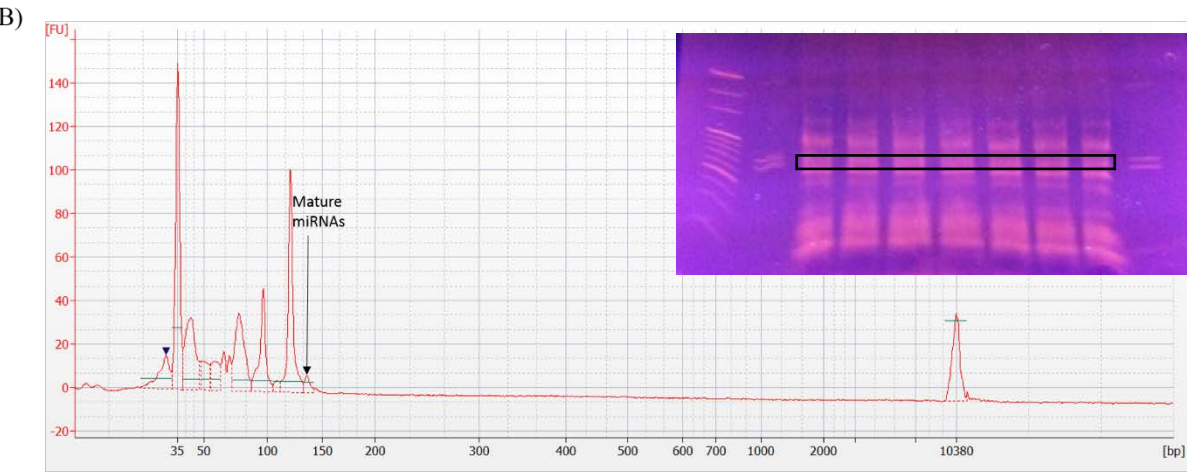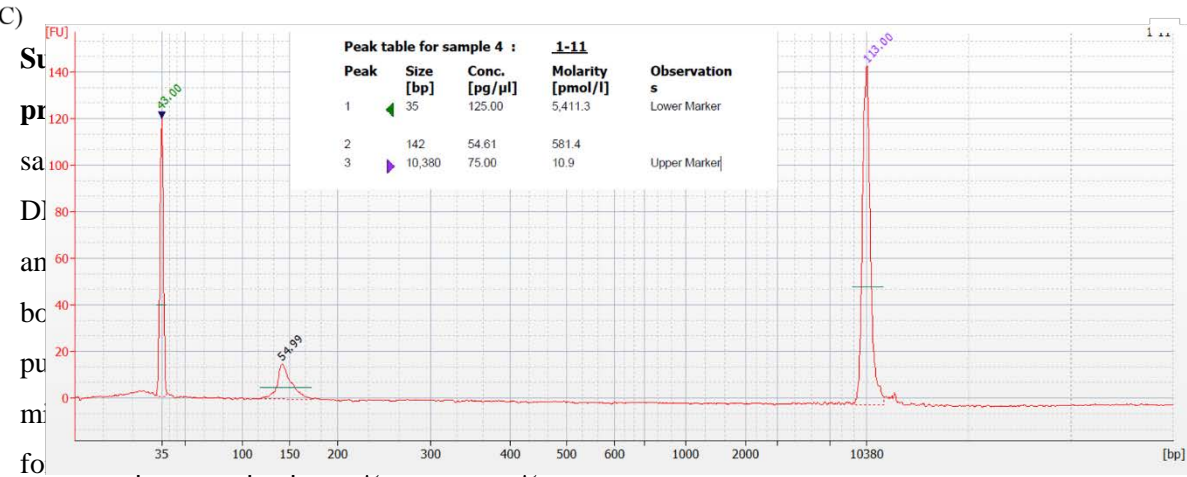

Supplement: Supplementary Figure 1 — Quality control testing of CSF small RNA sequencing library preparations. (A) A typical small RNA bioanalyzer trace of a miRVana PARIS extracted CSF sample (red, approximately 22nt) aligned with the small RNA ladder (blue). (B) A High sensitivity DNA bioanalyzer chip trace showing the CSF cDNA amplicon after 15 cycles of PCR amplification and the gel insert identifying the corresponding miRNA band in each sample (black box) during library preparation. (C) A High sensitivity DNA bioanalyzer chip trace showing the purified amplified CSF cDNA library construct, the 142 bp peak primarily containing mature microRNA generated from approximately 22 nt small RNA fragments. This sample is now ready for subsequent sample pooling and cluster generation. [file Image1.pdf]

## Supplementary Figure 2

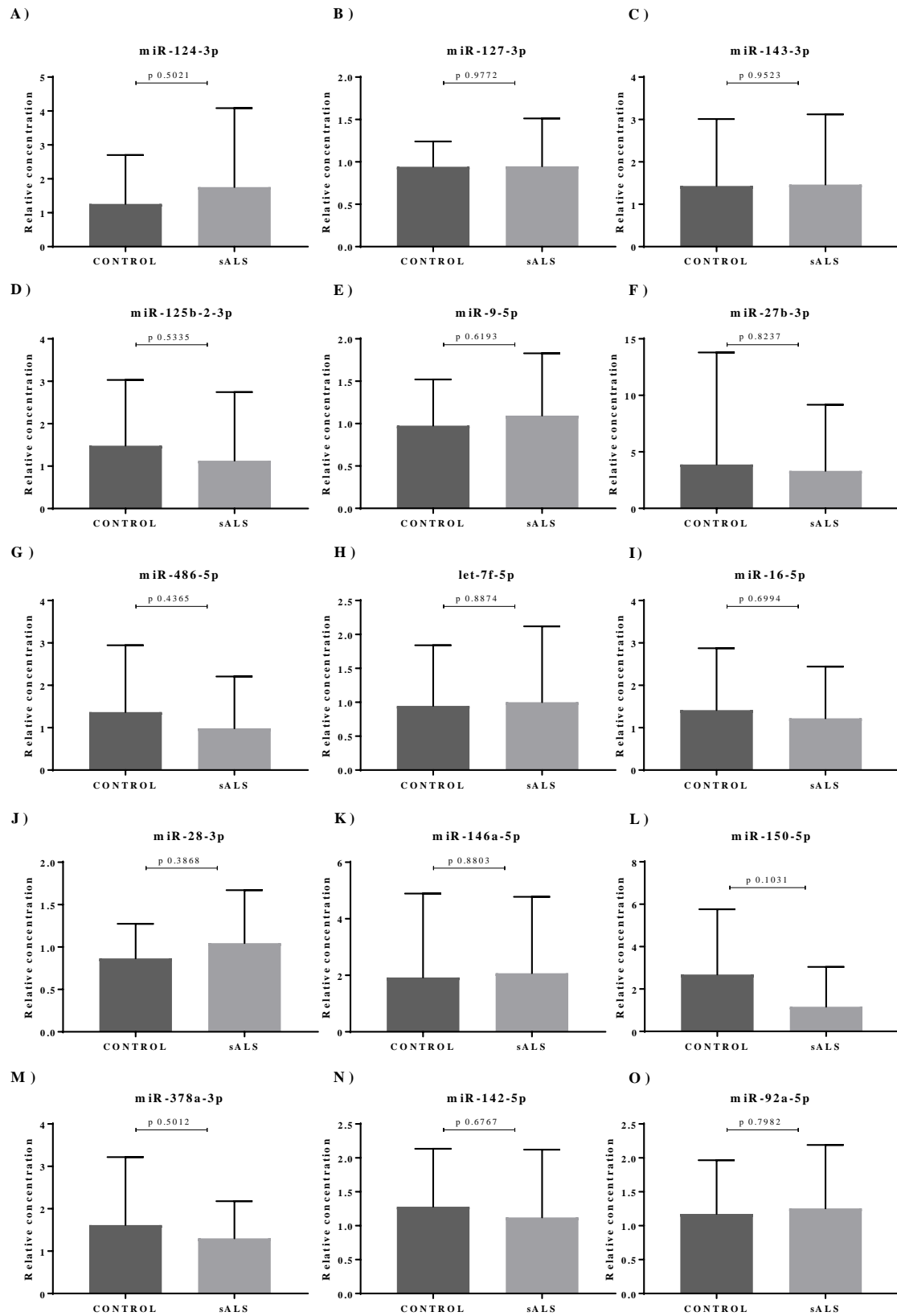

Supplement: Supplementary Figure 2 — miRNA qPCR validation. From the candidate miRNAs identified as statistically significantly expressed in sALS patients using small RNA sequencing, 7/15 miRNAs showed a non-significant same directional change when investigated using qPCR (A,E,G,I,L–N). The remaining 8/15 miRNAs showed very little change between sALS patients and control subjects using qPCR (B–D,F,H,J,K,O). [file Image2.pdf]
